# Microbial hallmarks of the respiratory tract in lung cancer: a meta-analysis

**DOI:** 10.3389/frmbi.2025.1589686

**Published:** 2025-07-01

**Authors:** Bin Zhu, Stephanie S. McHale, Michelle Van Scoyk, Gregory Riddick, Pei-Ying Wu, Chu-Fang Chou, Katherine Y. Tossas, Ching-Yi Chen, Robert A. Winn

**Affiliations:** ^1^ Massey Comprehensive Cancer Center, Virginia Commonwealth University, Richmond, VA, United States; ^2^ Department of Social and Behavioral Sciences, School of Public Health, Virginia Commonwealth University, Richmond, VA, United States; ^3^ Department of Epidemiology, School of Public Health, Virginia Commonwealth University, Richmond, VA, United States; ^4^ Center for Microbiome Engineering and Data Analysis, Virginia Commonwealth University, Richmond, VA, United States

**Keywords:** lung cancer, lung microbiota, microbiome, bronchoalveolar lavage, oral microbiota

## Abstract

**Introduction:**

Lung cancer is a leading cause of cancer-related deaths and has been associated with the microbiota of the human respiratory tract. However, the optimal sample type for studying the role of microbiota in lung cancer and the microbial hallmarks of lung cancer patients remain unclear.

**Methods:**

In this study, we downloaded 16S rRNA sequencing data of 1,105 high-quality samples from 13 BioProjects, including lung tissues, bronchoalveolar lavage (BAL) fluids, and saliva, and performed a meta-analysis.

**Results:**

Our results revealed that the BAL microbiota, dominated by taxa such as *Sphingomonas* and *Pseudomonas*, which are not typically abundant in the oral microbiota, served as hallmarks of individuals without lung cancer. In contrast, BAL samples from lung cancer patients showed higher relative abundances of oral-associated taxa, e.g., *Streptococcus* and *Prevotella*, with increased rates of dominance by these taxa in the BAL microbiota of lung cancer patients. Additionally, beta diversity analysis revealed significant compositional differences between the BAL microbiota of healthy individuals and those with lung cancer. Furthermore, while compositional differences were observed in the oral microbiota between healthy participants and lung cancer patients, as well as between microbiota from lung tumors and normal adjacent tissues, these differences were less pronounced than those observed in the BAL samples between healthy individuals and lung cancer patients. Cross-site correlations indicated limited associations between the relative abundances of taxa in the oral, BAL, and lung tissue microbiota, implying that differences in lower respiratory microbiota may not be directly driven by upper respiratory tract microbiota.

**Discussion:**

These findings highlight distinct microbial patterns linked to lung cancer in the respiratory tract. More pronounced differences were observed in the BAL microbiota between healthy individuals and lung cancer patients, with the predominance of taxa, typically not abundant in the oral microbiota, serving as hallmarks of health.

## Introduction

Lung cancer is the most prevalent and deadly cancer worldwide (Bray et al., 2024). In 2022, lung cancer was responsible for over 1.82 million deaths globally, representing approximately 18.7% of all cancer-related fatalities. Early-stage lung cancer often presents without obvious symptoms, leading to diagnoses that typically occur only after the cancer has metastasized or reached an advanced stage (Nooreldeen and Bach, 2021). This delayed diagnosis limits treatment options and contributes to a poor prognosis. Therefore, early detection is critical for improving survival outcomes for lung cancer patients.

In recent years, the rapid advancement of molecular biology techniques, particularly sequencing technologies, has positioned microbiota analysis as a promising approach for early cancer detection (Bagheri et al., 2022). One widely used method in respiratory microbiome research is 16S ribosomal RNA (16S rRNA) sequencing, which allows for the identification and analysis of bacterial communities within a microbiome. Changes in the composition of the human respiratory microbiota have been associated with a variety of diseases, including asthma, chronic obstructive pulmonary disease, cystic fibrosis, and lung cancer (Mao et al., 2018; Bou Zerdan et al., 2022). Studies on the human respiratory microbiota typically utilize saliva or oral swabs for analyzing the oral microbiota, bronchoalveolar lavage fluid (BAL) for the bronchoalveolar microbiota, and lung tissue samples for the lung microbiota (Mao et al., 2018). A BAL sample is obtained by introducing a small amount of sterile fluid into a specific area of the lung through a bronchoscope and then collecting the fluid for analysis. While many previous studies have employed bronchoscopy samples to investigate the lung microbiota, our study distinguishes between BAL and lung tissue samples to provide a more nuanced understanding of microbial communities.

In microbiota studies, alpha diversity describes the variety of microbial species within a single sample, reflecting both the number of species and their relative abundance distribution, while beta diversity compares the differences in microbial composition between samples, showing how similar or distinct the communities are. In two independent studies, lung cancer patients exhibited lower alpha diversity in their oral microbiota (Hosgood et al., 2021; Vogtmann et al., 2022). Although the difference in alpha diversity was statistically significant, the 95% confidence interval was relatively narrow, ranging from 0.84 to 0.96, indicating a substantial but low level of difference. One study identified *Streptococcus* as a risk factor for lung cancer (Vogtmann et al., 2022), while another reported the Bacilli class and Lactobacillales order as risk factors, and associated *Spirochaetia* and *Bacteroidetes* with a reduced risk of lung cancer (Hosgood et al., 2021). Similarly, lower alpha diversity was observed in lung tumor tissues compared to normal adjacent tissues in lung cancer patients (Yu et al., 2016) and higher levels of *Streptococcus* were linked to lung cancer (Liu et al., 2018). Another study further demonstrated that *Streptococcus intermedius* was isolated exclusively from the BAL samples of non-small-cell lung cancer patients (Sun et al., 2023), whereas a greater abundance of *Staphylococcus* was observed in control samples (Liu et al., 2018). Nevertheless, both oral and lung microbiota studies have limitations, including small sample sizes and inconsistent biomarker findings across studies (Mao et al., 2018; Zhang et al., 2023). Therefore, large-scale studies are needed to conduct more robust statistical analyses and validate these associations.

Although several bacterial taxa in the respiratory microbiota are known to be associated with lung cancer, the underlying mechanisms remain unclear. Various hypotheses have been proposed regarding the potential interactions between microbes and lung cancer. Under healthy conditions, the lung microbiome of healthy individuals maintains a dynamic equilibrium that supports normal physiological functions (Pérez-Cobas et al., 2023). However, dysbiosis of the lung microbiome may contribute to lung cancer development through several mechanisms (Natalini et al., 2023; Pérez-Cobas et al., 2023; Belaid et al., 2024). The enrichment of specific bacterial species in the lungs of lung cancer patients may trigger persistent chronic inflammatory responses, leading to continuous tissue damage and repair, thereby increasing the risk of cancer development. Chronic inflammation is a well-established risk factor for cancer, as it promotes DNA damage and abnormal cell proliferation through the release of pro-inflammatory mediators, free radicals, and immune-suppressive agents. Additionally, alterations in the lung microbiome have the potential to modify the host immune response, facilitating tumor immune evasion (Natalini et al., 2023; Pérez-Cobas et al., 2023; Belaid et al., 2024). Thus, the imbalance of the lung microbiome may play a critical role in lung cancer development by disrupting immune responses, promoting chronic inflammation, and fostering conditions favorable for tumor growth.

Bacterial biomass in the lower respiratory tract is generally lower than in the upper respiratory tract, largely due to effective microbial clearance mechanisms such as coughing, mucociliary transport, and immune responses (Natalini et al., 2023; Pérez-Cobas et al., 2023). However, these clearance functions may be compromised in lung cancer patients (Mazzoccoli et al., 2003; Prado-Garcia et al., 2012; Tilley et al., 2015). Consistent with this, lung cancer patients have been shown to exhibit a higher bacterial load in the lower respiratory tract compared to individuals without cancer (Leng et al., 2021). Thus, another hypothesis is that impaired microbial clearance in the lower respiratory tract contributes to alterations in the lung microbiota observed in lung cancer patients (Natalini et al., 2023; Pérez-Cobas et al., 2023).

In this study, publicly available data on the oral, BAL, and lung tissue microbiota were collected and our meta-analysis compared the oral and BAL microbiota between healthy participants and lung cancer patients as well as the microbiota between tumor and normal adjacent tissues in lung cancer patients. Microbes and diversities of the microbiota significantly associated with lung cancer in the three types of samples were identified. The BAL microbiota, dominated by taxa that are not typically abundant in the oral microbiota, served as hallmarks of individuals without lung cancer. Furthermore, since more significant differences were observed in the BAL microbiota than in the oral microbiota when comparing healthy individuals and lung cancer patients, and because collecting lung tissue samples from healthy individuals is challenging in clinical settings, BAL may represent a more suitable sample type for studying microbiota associated with lung cancer.

## Methods

### Search strategy

BioProjects were searched in the Sequence Read Archive (SRA) database (https://www.ncbi.nlm.nih.gov/sra) using a strategy that incorporated four key terms: (microbiota OR microbiome) AND (16S rRNA) AND (lung OR oral OR BAL OR bronchoalveolar lavage) AND (lung cancer). Given the limited number of samples from healthy participants in lung cancer research, an additional search was conducted with the following keywords: (microbiota OR microbiome) AND (16S rRNA) AND (lung OR BAL OR Bronchoalveolar lavage) AND (control OR healthy).

### Inclusion and exclusion criteria

The inclusion criteria were as follows: 1) Raw 16S rRNA sequencing data were publicly available for download from the SRA; 2) The health status of participants, such as healthy control, lung cancer, or adenocarcinoma, was clearly specified; 3) Only microbiota from three body niches, i.e., oral, BAL, and lung, were included; 4) Participants were not restricted by lung cancer subtype, sex, race, stage, or smoking history; 5) The study was an original research article. The exclusion criteria included: 1) Studies not related to lung cancer, except those involving BAL samples from healthy participants; 2) Studies with fewer than 20 samples, although all BAL samples from healthy participants were included without a sample size threshold for each study; 3) Participants under the age of 18 were excluded.

### Data preprocessing

After quality control, trimming, merging paired sequence reads, and removing human reads, high-quality sequences of the 16S rRNA amplicons were aligned to the 16S rRNA database as previously described (Zhu et al., 2024) using the ublast tool (Edgar, 2010) with -id, -query_cov, and -evalue set as 0.97, 0.9, and 1e-5, respectively. Briefly, the 16S rRNA database was created based on the Greengenes database version gg_13_5 (https://greengenes.secondgenome.com/) and the HOMD database version 15.1 (https://www.homd.org/) (DeSantis et al., 2006; Chen et al., 2010). Due to the inability to annotate many taxa at the species level and the low relative abundances of many species-level taxa, taxonomic annotation was performed at the genus level in this study. Only taxa with a relative abundance of at least 0.1% (or 0.01%) in at least 5% (or 15%) of the samples were retained in the feature table of the 16S rRNA profiles, as previously described (Zhu et al., 2024). Samples with a total read count of less than 2,000 were excluded from the analysis, resulting in a final dataset of 1,105 samples.

### Clustering of microbiota samples

The relative abundance of taxa in each microbiota was used for clustering analysis. Specifically, the ‘pheatmap’ function was utilized with the parameters ‘clustering_distance_cols’ set to ‘manhattan’ and ‘clustering_method’ set to ‘ward.D2’.

### Alpha and beta diversity

The feature table of the 16S rRNA profiles was normalized by rarefaction to the sequencing depth of the sample with the fewest reads (>2,000). Alpha diversity was quantified using the Shannon index, calculated with the ‘vegan’ package in R (Jari Oksanen et al., 2020). The difference in alpha diversity between groups was assessed using the Wilcoxon test. Beta diversity was evaluated using Bray-Curtis dissimilarity, also with the ‘vegan’ package in R. The difference in beta diversity between two groups was tested using the ‘adonis2’ function, a PERMANOVA analysis, within the ‘vegan’ package in R. To identify the influence of multiple factors on the composition of microbiota, the adonis test with a marginal model was applied using the parameter ‘by’ set as “margin”.

### Differential abundance analysis

Differential abundance analysis was conducted using the ‘ALDEx2’ package in R (Fernandes et al., 2013). The adjusted P-value for relative abundance differences was calculated using the ‘aldex.ttest’ function, which applied the two-sided Mann-Whitney U test, followed by the Benjamini-Hochberg correction. The relative abundance change was measured using the ‘aldex.effect’ function, quantified by the per-feature median difference between two conditions. For analyses in which no significant differences were detected, differential abundance analysis was performed using LefSe analysis through the ‘run_lefse’ function in the microbiomeMarker package in R.

### Removal of batch effect

For BAL samples, batch effects across cohorts were corrected using the ‘adjust_batch’ function in the ‘MMUPHin’ package (Ma et al., 2022) in R with BioProject set as the batch and lung cancer status as a covariate. For lung tissue samples, batch effects across cohorts were corrected using the ‘MBECS’ package in R (Olbrich et al., 2023). Specifically, the ‘mbecCorrection’ function was applied with the parameters ‘method’ set to ‘rbe’ and ‘type’ set to ‘clr’ to address batch effects.

### Correlation analysis among taxa between two microbiota

Spearman’s correlation was used to evaluate the relationship between taxa in two microbiota. The correlation coefficient (R-value) and its significance (P-value) were calculated, with P-values adjusted using the Benjamini-Hochberg procedure via the ‘adjust.p’ function from the ‘cp4p’ package in R. In heatmap visualizations, R-values associated with adjusted P-values greater than 0.05 were replaced with zeros to exclude insignificant correlations. Taxa were clustered in the heatmaps based on the R-values of the Spearman’s correlation using the ‘pheatmap’ function with default settings in R.

## Results

### Profiles of the respiratory microbiota

A total of 1,385 samples originated from 13 BioProjects (**Supplementary Figure S2a**) (Rylance et al., 2016; Yu et al., 2016; Kovaleva et al., 2020; Nejman et al., 2020; Zhuo et al., 2020; Bello et al., 2021; He et al., 2022; Guo et al., 2023; Najafi et al., 2023) were downloaded from the SRA database and included in this study (**Supplementary Figure S1a**), with inclusion criteria detailed in the Methods section. To determine an appropriate total reads threshold for individual samples, alpha rarefaction analysis was performed across various rarefaction depths. As illustrated in **Supplementary Figure S1b**, the number of observed taxa at the genus level did not differ significantly between rarefaction depths of 1,800 and 2,000, indicating that samples with fewer than 2,000 total reads had limited impact on diversity estimates. Consequently, samples with fewer than 2,000 total reads were excluded, resulting in 1,105 samples retained for analysis. Additionally, taxa with low relative abundance or those that appeared infrequently across the 1,105 samples were removed from the taxonomic profiles (see the Methods), leaving 416 taxa at the genus level for subsequent analyses. Given that many taxa were not annotated at the species level, taxonomic classification at the genus level was used throughout the study.

The 1,105 high-quality samples spanned 8 countries (**Supplementary Figure S2b**). The sample distribution included 594 lung samples, 425 BAL samples, and 86 saliva samples (**Supplementary Figure S2c**). All oral microbiota samples in this study were derived from saliva, not oral swabs. Sputum, though another common respiratory sample type, contains a mix of microbes from both the upper and lower respiratory tracts. To ensure specific site analysis, sputum was excluded from this study. Of the 594 lung samples, 212 were from primary lung tumors, while the remainder were derived from non-malignant lung tissues or tumor-adjacent lung tissues; none were collected from healthy participants. Various lung cancer types, primarily adenocarcinoma and squamous cell carcinoma, were represented (**Supplementary Figure S2d**), although the specific cancer subtype was unspecified in some samples. Approximately half of the total samples contained demographic information on age, race, sex, and smoking status. Most participants were between 40 and 80 years old (**Supplementary Figure S2e**). About 84% of the total samples came from non-Hispanic white individuals (**Supplementary Figure S2f**), 70% were from males (**Supplementary Figure S2g**), and 7.2% were from current smokers (**Supplementary Figure S2h**).

### Association between microbiota in bronchoalveolar lavage fluid and lung cancer

Of the nine datasets containing BAL samples, only one (PRJNA586753) included samples from both lung cancer patients and healthy participants (**Supplementary Figure S3a**), while the other datasets contain samples from either healthy individuals or lung cancer patients exclusively. Consequently, three analytical strategies were employed to address the limitations posed by this unbalanced study design.

**Figure 1:**
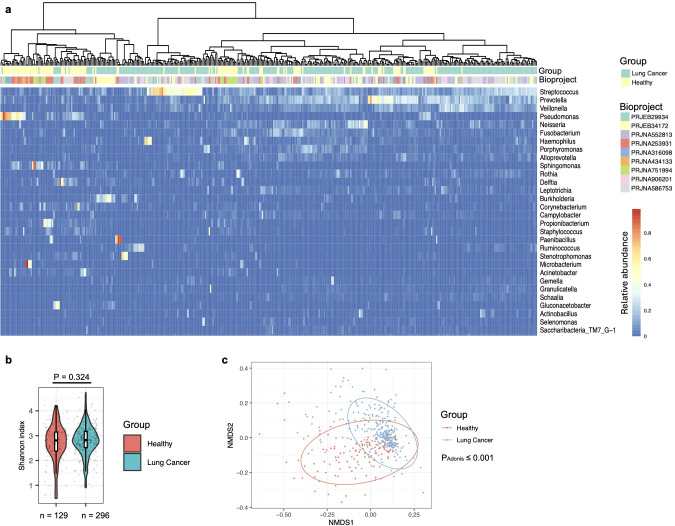
Composition of the bronchoalveolar lavage (BAL) fluids microbiota in healthy individuals and lung cancer patients. **(a)** Lung cancer status and BioProject IDs are indicated by color-coded labels at the top of the heatmap. Samples are clustered using the “ward.D2” method with Manhattan distance. **(b)** Alpha diversity of the BAL microbiota, quantified by the Shannon index, is compared between healthy and lung cancer participants, with differences assessed using the two-sided Mann-Whitney U test. The case numbers are indicated below the box plot. **(c)** The relationship between lung cancer and BAL microbiota composition is visualized in an NMDS plot and tested by the Adonis test with default parameters.

The first strategy was to perform a batch effect removal prior to further analysis with BioProject set as the batch and lung cancer status as a covariate (see Methods). As described previously (Natalini et al., 2023) and observed in this study (**Supplementary Figure S4**), *Streptococcus*, *Prevotella*, *Veillonella*, *Rothia*, *Porphyromonas*, and *Neisseria* were the predominant taxa in the oral microbiota. After batch correction, most BAL microbiota from healthy participants clustered on the left side of the heatmap, indicating similar microbial profiles among these samples (**Figure 1a**). Furthermore, many BAL microbiota samples from healthy participants, e.g., those predominated by *Sphingomonas*, *Pseudomonas*, *Propionibacterium*, *Fusobacterium*, and *Microbacterium*, were not dominated by taxa predominant in the oral cavity (**Figure 1a**, **Table 1**; **Supplementary Dataset 1 Sheet 1**). This suggests that BAL microbiota characterized by the predominance of taxa not abundant in the oral cavity serves as a hallmark of individuals without lung cancer. Additionally, several taxa frequently observed as the most abundant in the BAL microbiota of lung cancer patients, including *Neisseria*, *Veillonella*, and *Porphyromonas*, were rarely detected at high abundance in samples from healthy participants (**Supplementary Dataset 1 Sheet 1**). *Streptococcus* and *Prevotella* were abundant in both the oral and BAL microbiota, but their prevalence as predominant taxa was significantly lower in the BAL microbiota (**Table 1**). Consistent with these observations, differential abundance analysis showed that oral-abundant taxa, e.g., *Streptococcus*, *Prevotella*, *Veillonella*, *Rothia*, *Porphyromonas*, and *Neisseria*, were more abundant in the BAL microbiota of lung cancer patients, while taxa not abundant in the oral microbiota, e.g., *Sphingomonas* and *Pseudomonas*, were enriched in the BAL microbiota of healthy individuals (**Supplementary Dataset 1, Sheet 2**).

**Table 1:** The number of BAL samples predominant by a specific taxon in healthy and lung cancer participants.

Most abundant taxon	Number of BAL samples predominant by this taxon in healthy participants	Number of BAL samples predominant by this taxon in lung cancer patients	Odds_Ratio	Lower_CI	Upper_CI	Fisher P-value
*Streptococcus*	8	81	5.69825581	2.66554476	12.1814196	1.39E-07
*Prevotella*	23	80	1.70692432	1.01610875	2.86740039	0.04868917
*Pseudomonas*	23	2	0.03135167	0.00726741	0.13525133	4.69E-11
*Propionibacterium*	8	0	0	0	NA	6.17E-05
*Sphingomonas*	6	0	0	0	NA	0.00072006
*Corynebacterium*	5	0	0	0	NA	0.0024389
*Fusobacterium*	9	4	0.1826484	0.05518825	0.60448447	0.00385898
*Novosphingobium*	3	0	0	0	NA	0.02751112
*Microbacterium*	4	1	0.1059322	0.01172276	0.95725178	0.0313155

The full list is provided in **Supplementary Dataset 1**. The numbers of samples from healthy individuals and lung cancer patients were 129 and 296, respectively.

Diversity analyses showed that alpha diversity of the BAL microbiota, quantified at the genus level using the Shannon index, was similar between healthy individuals and lung cancer patients (**Figure 1b**). Supporting the clustering pattern observed in **Figure 1a**, the Non-metric Multidimensional Scaling (NMDS) plot, along with a PERMANOVA analysis (Adonis test), confirmed that the composition of the BAL microbiota was significantly associated with lung cancer status (**Figure 1c**).

As a second strategy to address the unbalanced cohort design, an Adonis test was conducted incorporating multiple independent variables, i.e., lung cancer status, country, BioProject, sex, age, stage, race, and smoking status, using a marginal model. Each variable was tested independently of order, while controlling for all other variables in the model. Therefore, the independent effect of lung cancer status on the microbiota was assessed. As shown in **Supplementary Dataset 2**, lung cancer was the second most influential factor before batch effect removal and became the most influential factor after correction. Additionally, BioProject, age, smoking, and country were also significantly associated with variation in the BAL microbiota following batch correction, independent of the other factors in the analysis.

**Figure 2:**
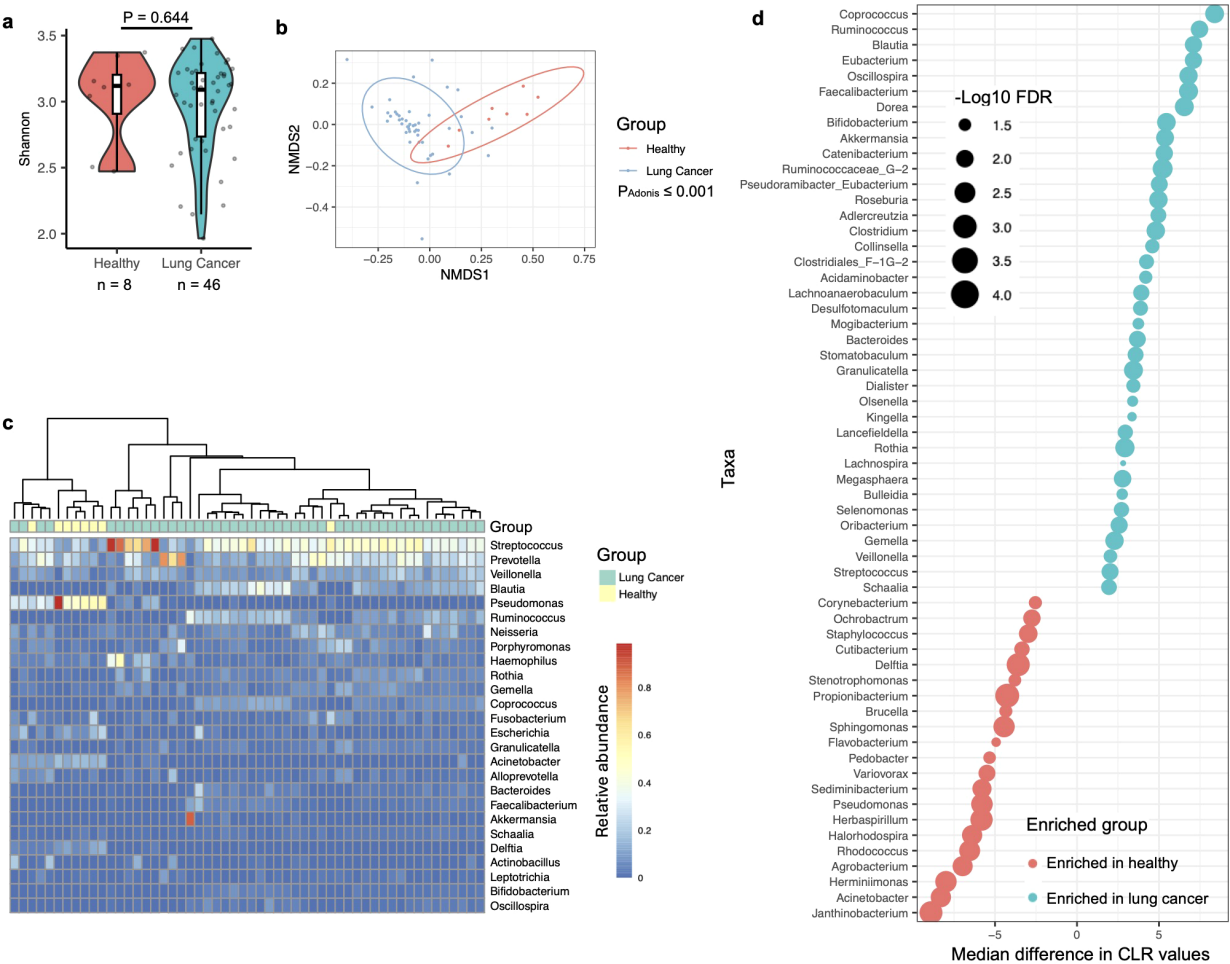
Association of the BAL microbiota with lung cancer analyzed by data from the PRJNA586753 cohort. **(a)** Alpha diversity of the BAL microbiota, quantified by the Shannon index, is compared between healthy and lung cancer participants, with differences assessed using the two-sided Mann-Whitney U test. The case numbers are indicated below the box plot. **(b)** The relationship between lung cancer and BAL microbiota composition is visualized in an NMDS plot and tested by the Adonis test with default parameters. **(c)** The composition of the BAL microbiota in healthy and lung cancer participants is shown in the heatmap, with samples clustered using the “ward.D2” method and Manhattan distance. **(d)** Relative abundance differences of bacterial taxa between healthy and lung cancer participants in the BAL microbiota are shown. Changes in relative abundance were tested using the ALDEx2 package in R, and quantified by the per-taxon median difference between conditions. Adjusted P-values were calculated with the Benjamini-Hochberg correction applied to the Mann-Whitney U test.

Given that the PRJNA586753 dataset contained BAL microbiota samples from both healthy individuals and lung cancer patients, the third strategy was to analyze this individual cohort in detail. Alpha diversity, measured by the Shannon index at the genus level, showed no significant association with lung cancer in this dataset (**Figure 2a**). However, beta diversity demonstrated an obvious compositional difference in the BAL microbiota between healthy individuals and lung cancer patients (**Figure 2b**). PERMANOVA analysis using the Adonis test further confirmed that BAL microbiota composition was significantly associated with lung cancer.

Consistent with observations across the nine datasets, a heatmap of the PRJNA586753 dataset revealed that BAL microbiota in healthy participants was predominantly composed of *Pseudomonas*, a genus not abundant in the oral cavity (**Figure 2c**) (Natalini et al., 2023). Differential abundance analysis using the ALDEx2 software indicated that taxa abundant in the oral cavity, i.e., *Streptococcus* and *Veillonella*, were significantly enriched in the BAL microbiota of lung cancer patients (**Figure 2d**). In contrast, taxa typically not abundant in the oral microbiota, particularly, *Sphingomonas* and *Pseudomonas* (**Supplementary Dataset 1**), were significantly enriched in the BAL microbiota of healthy participants.

Overall, these observations suggest a significant association between the composition of the BAL microbiota and lung cancer. Specifically, a BAL microbiota that is dominated by taxa not abundant in the oral cavity serves as a potential hallmark for lung health.

### Association between the oral microbiota and lung cancer

Since oral microbes are a primary source of the BAL microbiota, variations in the oral microbiota may underlie the differences in the BAL microbiota associated with lung cancer. To test this hypothesis, we analyzed the oral microbiota from saliva samples. Two datasets included saliva samples: one dataset (PRJEB29934) contained samples solely from lung cancer patients, while the other dataset (PRJNA586753) included samples from both healthy individuals and lung cancer patients. For our analysis, we focused on the PRJNA586753 dataset.

**Figure 3:**
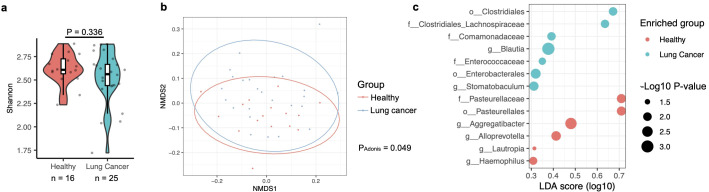
Association of the oral microbiota with lung cancer, based on data from the PRJNA586753 cohort. **(a)** Alpha diversity of the oral microbiota, measured by the Shannon index, compared between healthy participants and those with lung cancer. Differences were evaluated using the two-sided Mann-Whitney U test, with case numbers indicated below the box plot. **(b)** The relationship between lung cancer and oral microbiota composition is illustrated in an NMDS plot and analyzed using the Adonis test with default parameters. **(c)** Differences in the relative abundance of bacterial taxa in the oral microbiota between healthy and lung cancer participants are shown. Relative abundance changes were assessed with LefSe analysis in R, and quantified by the LDA score across conditions.

The analysis methods were consistent with those described above. At the genus level, there was no significant difference in the oral microbiota associated with lung cancer (**Figure 3a**). However, the Adonis test revealed that the overall composition of the oral microbiota was significantly associated with lung cancer (**Figure 3b**), although the P-value for this difference (P = 0.049) was notably weaker than that observed for the BAL microbiota (P ≤ 0.001), despite comparable case numbers (**Figures 2a**, **3a**). A differential abundance analysis using ALDEx2 was also conducted, but no taxa displayed significantly different abundances related to lung cancer (data not shown). To explore potential differences further, a less stringent method, LefSe (Segata et al., 2011), was applied, identifying enrichment of the genera *Blautia* and *Stomatobaculum* in lung cancer patients and *Aggregatibacter*, *Alloprevotella*, *Lautropia*, and *Haemophilus* in healthy participants (**Figure 3c**). Notably, *Blautia* and *Stomatobaculum* were also enriched in lung cancer patients within the oral microbiota (**Figure 2d**), although their significance was stronger in the BAL samples. However, the four genera associated with health in the oral microbiota were not identified as differentially abundant in the BAL microbiota after correcting for multiple testing.

Consistent with previous studies (Hosgood et al., 2021; Vogtmann et al., 2022; Zhang et al., 2023), the results described above indicate a significant difference in the composition of the oral microbiota between healthy individuals and those with lung cancer, although this difference is less pronounced than that observed in the BAL microbiota. Given that *Blautia* and *Stomatobaculum* were both identified as potential risk factors for lung cancer, it is possible that the differences observed in the BAL microbiota are related to those in the oral microbiota. To further test this hypothesis, we analyzed the correlation of taxa in the oral and BAL microbiota using saliva and BAL samples case-matched by the same participants from the PRJNA586753 cohort, which includes samples from both healthy and lung cancer participants. To determine whether the relative abundance of microbes in the oral microbiota influences that in the BAL microbiota, Spearman’s correlations were calculated between specific bacterial taxa in the oral microbiota and their corresponding abundance in the BAL microbiota. Consistent with previous studies reporting significant difference between the oral and BAL microbiota (Bingula et al., 2020; Leitao Filho et al., 2023), the analysis revealed that only two out of 416 genera showed significant correlations in relative abundance between the oral and BAL samples, and neither of these genera were associated with lung cancer (**Supplementary Figure S5**, **Supplementary Dataset 3**). These findings suggest that the relative abundance of microbes in the oral samples does not directly influence that in the microbiota in the BAL.

### The lung microbiota in lung cancer

For this study, five datasets containing lung microbiota data collected from tumor and normal adjacent tissue of lung cancer patients were analyzed (**Supplementary Figure S6a**). As no studies included lung tissue from healthy individuals, all comparisons below are between tumor and adjacent normal tissues from lung cancer patients. Additionally, one cohort (PRJNA327258) only contained tumor tissue samples and was excluded from the analysis. Since the remaining datasets contain both tumor and normal adjacent tissue samples, a batch effect removal procedure was applied to remove differences between these cohorts (**Supplementary Figures S6b,c**).

**Figure 4:**
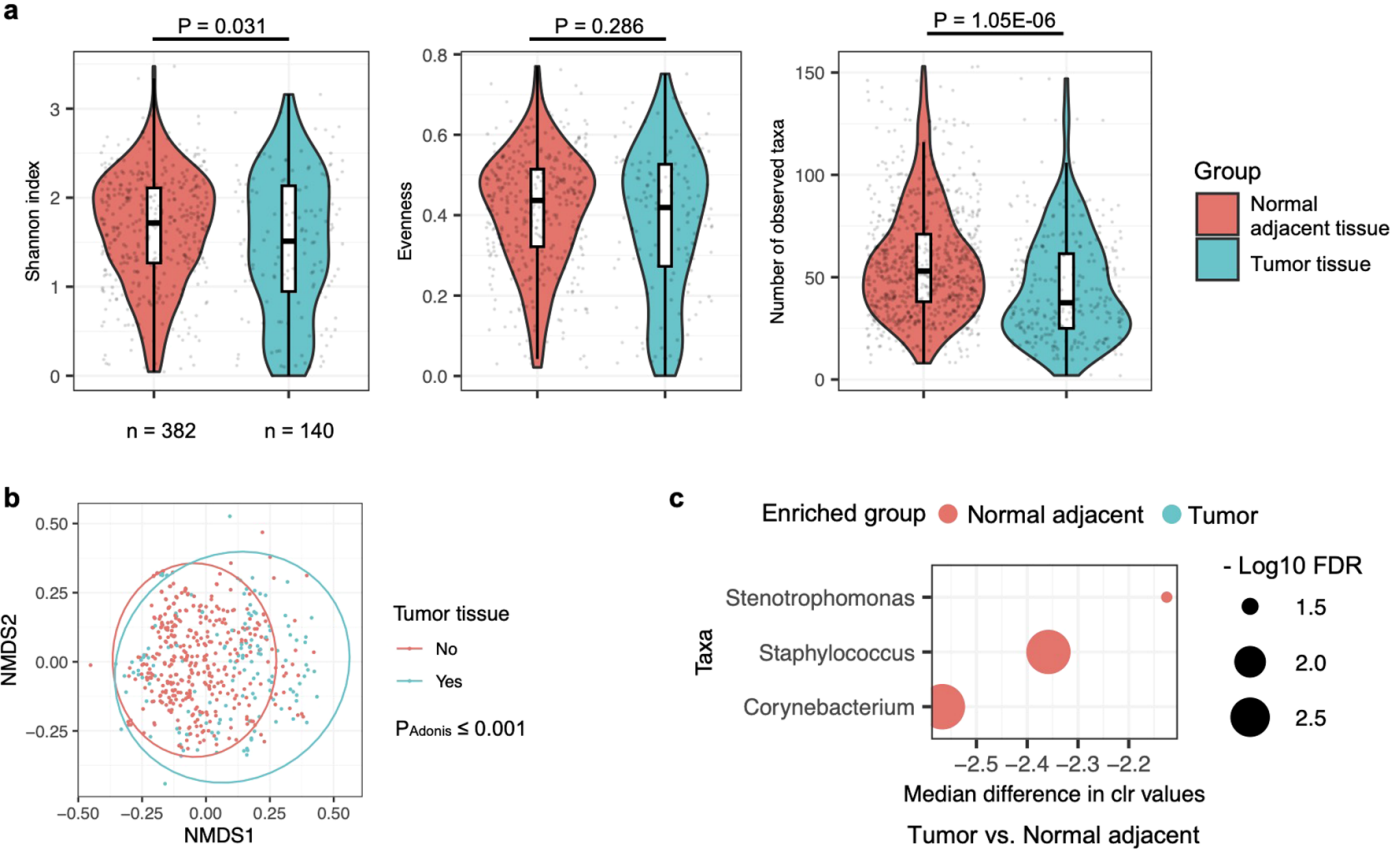
Comparison of microbiota in normal adjacent and tumor tissues in lung cancer patients. **(a)** Alpha diversity of the lung tissue microbiota, assessed by the Shannon index, evenness, and observed taxa count, is compared between normal adjacent and tumor tissues, with differences evaluated by the two-sided Mann-Whitney U test. Case numbers are displayed below the box plot. **(b)** The relationship between lung cancer and lung tissue microbiota composition is represented in an NMDS plot and analyzed by the Adonis test with default settings. **(c)** Differences in the relative abundance of bacterial taxa in the lung tissue microbiota between normal adjacent and tumor tissues are shown. Changes in relative abundance were evaluated using the ALDEx2 package in R, with quantification by the per-taxon median difference between conditions. Adjusted P-values were calculated using the Benjamini-Hochberg correction for the Mann-Whitney U test.

Probably due to the larger sample size, a notable difference in alpha diversity at the genus level was observed in the lung microbiota between normal adjacent and tumor tissues, as measured by the Shannon index (**Figure 4a**). This contrasts with the findings in the saliva and BAL microbiota, where such differences were not observed. The Shannon index reflects both evenness and the number of observed taxa, where evenness refers to the uniformity of species represented within a community. In the lung tissue microbiota, the number of genera but not evenness was significantly different between normal adjacent and tumor tissues. Beta diversity, in addition, was significantly different between normal adjacent and tumor tissues (**Figure 4b**), suggesting distinct microbial compositions associated with lung cancer in the lung tissue microbiota. Further differential abundance analysis using ALDEx2 identified three taxa, i.e., *Stenotrophomonas*, *Corynebacterium*, and *Staphylococcus*, as significantly enriched in normal adjacent tissues (**Figure 4c**). Consistent with the BAL microbiota findings, the three taxa enriched in normal adjacent lung tissues were also more abundant in BAL samples from healthy participants (**Figure 2d**).

In hierarchical clustering, most of the BAL samples from healthy participants were grouped close to each other, indicating similar community profiles (**Figure 1a**). As shown in **Supplementary Figure S7**, the same hierarchical clustering analysis was performed on the lung tissue microbiota. However, no distinct clusters were observed that predominantly included samples from normal adjacent or tumor tissues. This, combined with the less significant P-values in the Adonis test for beta diversity (**Figure 4b** compared to **Figure 1c**) and differential abundance analyses (**Figure 4c** compared to **Supplementary Dataset 2 Sheet 2**), suggests that the differences between the microbiota in normal adjacent and tumor tissues from lung cancer patients are less pronounced than those observed between the BAL microbiota of healthy and lung cancer participants.

### Association between taxa at different body sites in the respiratory tract

The results shown above suggested that the BAL microbiota has the most significant association with lung cancer and it seems that, although microbes in the oral cavity are a primary source of the lung microbiota (Bagheri et al., 2022), there was no significant correlation between the relative abundance of specific bacterial taxa in the oral and BAL microbiota (**Supplementary Figure S5**). To further investigate microbial associations across different body sites, we analyzed microbiota from saliva, BAL, and lung tumor tissue samples case-matched by the same participants, using data from the PRJEB29934 cohort.

In this study, a total of 416 taxa were identified at the genus level. However, only four taxa within the oral microbiota showed significant self-association (FDR ≤ 0.05) with their counterparts in the BAL microbiota (**Supplementary Figure S8a**). Likewise, only two taxa were correlated between the oral and lung tissue microbiota, and no taxa were correlated between the BAL and lung tissue microbiota (**Supplementary Figures S8b,c**). These findings suggest that, although the upper respiratory tract may serve as a primary source for microbiota in the lower respiratory tract, the relative abundances of taxa across different respiratory tract sites are not significantly associated. Therefore, oral and BAL samples are not suitable proxies for investigating the relative abundance of bacterial taxa in lung tissue samples.

## Discussion

This study systematically examined the association between lung cancer and the microbiota across various sites in the respiratory tract, including the oral cavity, BAL, and lung tissue. The BAL microbiota showed the strongest association with lung cancer when comparing healthy and lung cancer participants. Since BAL samples are collected from the lower respiratory tract, specifically from the alveolar spaces, this provides a closer interaction with lung tissue cells compared to oral samples. Thus, microbes in BAL samples may have a more direct impact on the lung microenvironment. A more substantial microbiota difference may exist between lung tissues from healthy participants and lung cancer tissues. However, the difficulty of collecting lung tissue samples from healthy participants limits the ability to detect such differences. In lung cancer patients, microbiota may move between normal adjacent and tumor sites or be influenced by common factors within the lung, such as immune suppression or impaired respiratory clearance. This could result in less distinct differences between normal adjacent tissue and tumor-associated microbiota in lung cancer patients compared to microbiota differences between BAL samples from healthy participants and lung cancer patients. Furthermore, recent studies have incorporated lung microbiota data into predictive models for lung cancer screening (Chen et al., 2023; Zhou et al., 2024; Kashyap et al., 2025). These findings further underscore the potential clinical utility of BAL samples in lung cancer diagnosis, given the significant differences observed between BAL microbiota in healthy individuals and lung cancer patients.

Another key finding is that a BAL microbiota composition dominated by taxa not abundant in the oral microbiota is a hallmark of individuals without lung cancer. In contrast, taxa commonly found in the oral microbiota, such as the *Streptococcus* genus, were more abundant in the BAL of lung cancer patients. This shift may be attributed to lung cancer impairing respiratory clearance, thereby facilitating the translocation of microbes from the upper to the lower respiratory tract. However, this observation does not rule out the possibility that microbial invasion from the upper respiratory tract could actively contribute to lung cancer development by inducing chronic inflammation, as discussed above. Therefore, these data cannot determine whether the BAL microbiota merely reflects reduced lung function or actively contributes to cancer pathogenesis. To establish the causal relationship between changes in BAL microbiota and lung cancer, longitudinal studies are essential. Identifying whether these microbiota shifts occur prior to or during the early stages of lung cancer could provide valuable insights for early diagnosis and intervention.

Multiple factors contribute to the differences between the microbiota of the upper and lower respiratory tracts, including but not limited to distinct microenvironments such as oxygen levels, as well as microbial clearance mechanisms like coughing, mucociliary transport, and immune responses, as introduced above. In this study, the BAL microbiota showed a stronger association with lung cancer compared to the oral microbiota. Although few significant correlations were observed between specific bacterial taxa across different body sites, this does not rule out the possibility that microbial communities in the upper respiratory tract may influence those in the lower respiratory tract and impact lung cancer. Microbial seeding from the upper to lower respiratory tract is likely, as the oral cavity serves as the body’s gateway and is more directly influenced by external environmental factors. Addressing this question will require data with strain-level resolution.

Past reviews have identified limited sample sizes and inadequate control for confounding variables as key limitations in understanding the role of respiratory microbiota in lung cancer. This study partially addressed the sample size limitation but could not control for confounding factors due to a lack of available information. Given the observed differences in BAL microbiota between healthy and lung cancer participants, future studies should consider larger, well-matched cohorts with expanded BAL sampling to improve understanding of these associations.

Another major limitation of this study is that significant challenges in specimen collection, processing, storage, and 16S rRNA sequencing make reproducibility across multiple sites and cohorts difficult. Even with similar collection strategies, results can vary substantially between locations, independent of true differences in microbial taxa. Therefore, some of the analysis outcomes, e.g., those shown in **Figures 2**, **3**, are derived from a single cohort. Although batch effect removal techniques were applied in the rest analyses, potential biases arising from cohort diversity could still impact the conclusions.

Contamination is another potential factor that may have influenced the analyses in this study. Approximately half of the cohorts either have associated publications describing protocols to minimize contamination (Nejman et al., 2020; Zhuo et al., 2020; He et al., 2022; Guo et al., 2023), like the use of transbronchoendoscope in sample collection, or include mock samples to control for environmental contamination (PRJEB34172). In contrast, for the cohorts PRJEB29934, PRJNA253931, PRJNA316098, and PRJNA434133, no related publications are available to confirm whether contamination control measures were taken. Therefore, potential contamination in these cohorts could have influenced the conclusions regarding the BAL microbiota.

## Data Availability

Publicly available datasets were downloaded from the NCBI database (https://www.ncbi.nlm.nih.gov/). The BioProject IDs with data used in this study are listed below: PRJEB29934, PRJEB34172, PRJNA253931, PRJNA303190, PRJNA316098, PRJNA327258, PRJNA434133, PRJNA472758, PRJNA552813, PRJNA586753, PRJNA647170, PRJNA751994, and PRJNA906201.
