# Supplementary material for: Microbial hallmarks of the respiratory tract in lung cancer: a meta-analysis
Source: Front Microbiomes. 2025 Jul 1;4:1589686. doi: 10.3389/frmbi.2025.1589686 (PMC12993565; doi:10.3389/frmbi.2025.1589686)
Supplement: Supplementary file 1 [file Supplementaryfile1.pdf]

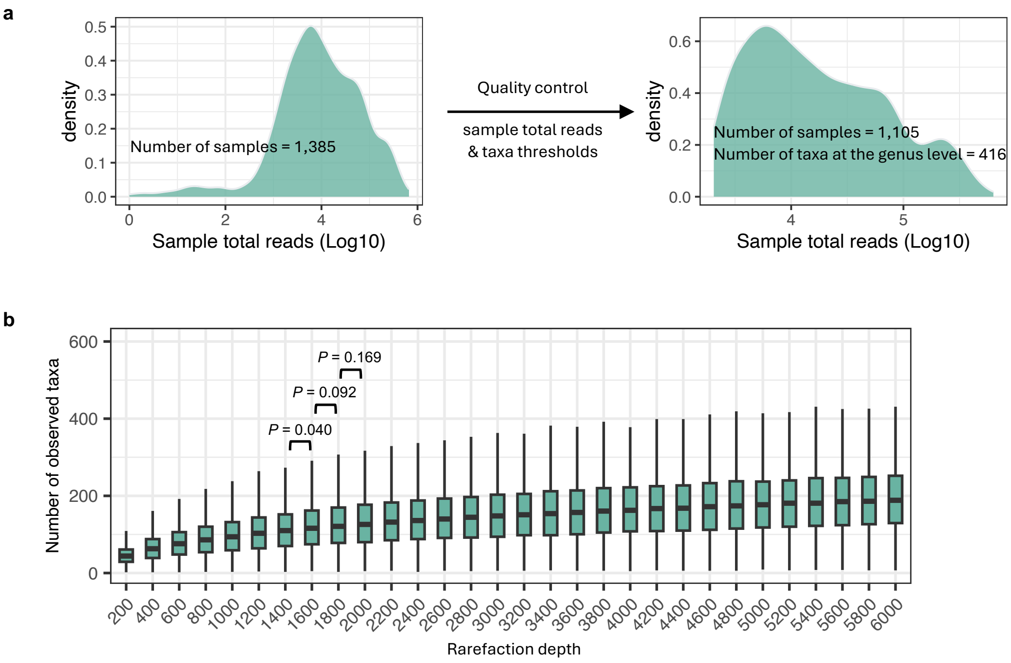

Fig. S1 Pre-treatment of microbiota profiles. (a) After quality control, trimming, merging of paired sequence reads, and removal of human reads, the total reads for 1,385 samples are displayed in the left panel. Applying the taxa and sample total reads threshold (as outlined in the Methods) retained 1,105 high-quality samples with 416 bacterial taxa at the genus level. **(b)** Alpha rarefaction of the 1,385 samples indicates that a total sample reads threshold of 2,000 is suitable for subsequent analyses. Differences between conditions were assessed using the two-sided Mann–Whitney U test.

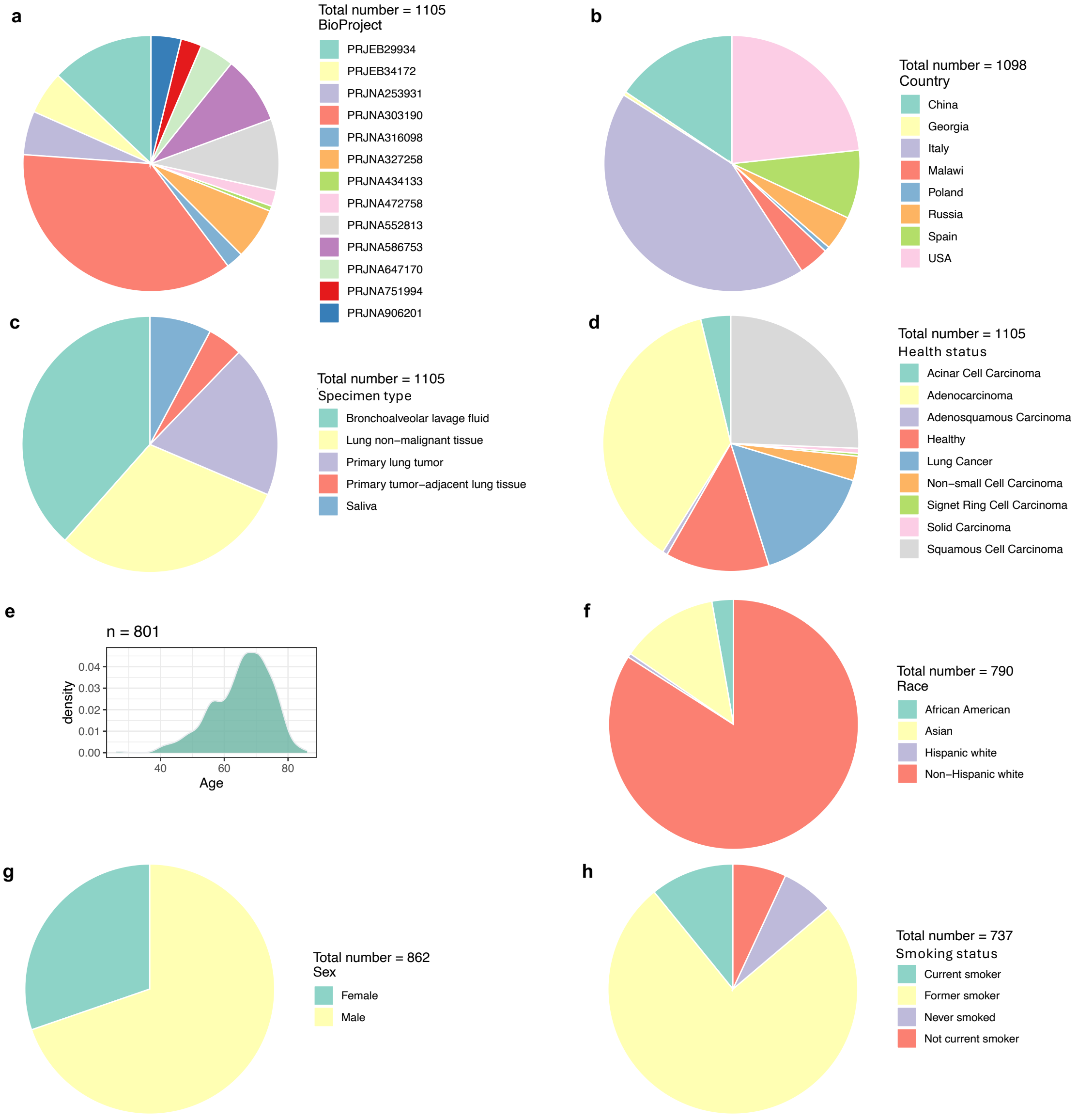

Fig. S2 Metadata profiles of samples used in this study. Profiles include BioProject **(a),** country of sample collection **(b),** specimen type **(c),** health status **(d),** participant age **(e),** race **(f),** sex **(g),** and smoking status **(h).** In the panel d, “heathy” indicates samples from healthy participants and “lung cancer” are those from lung cancer patients without defined subtypes.

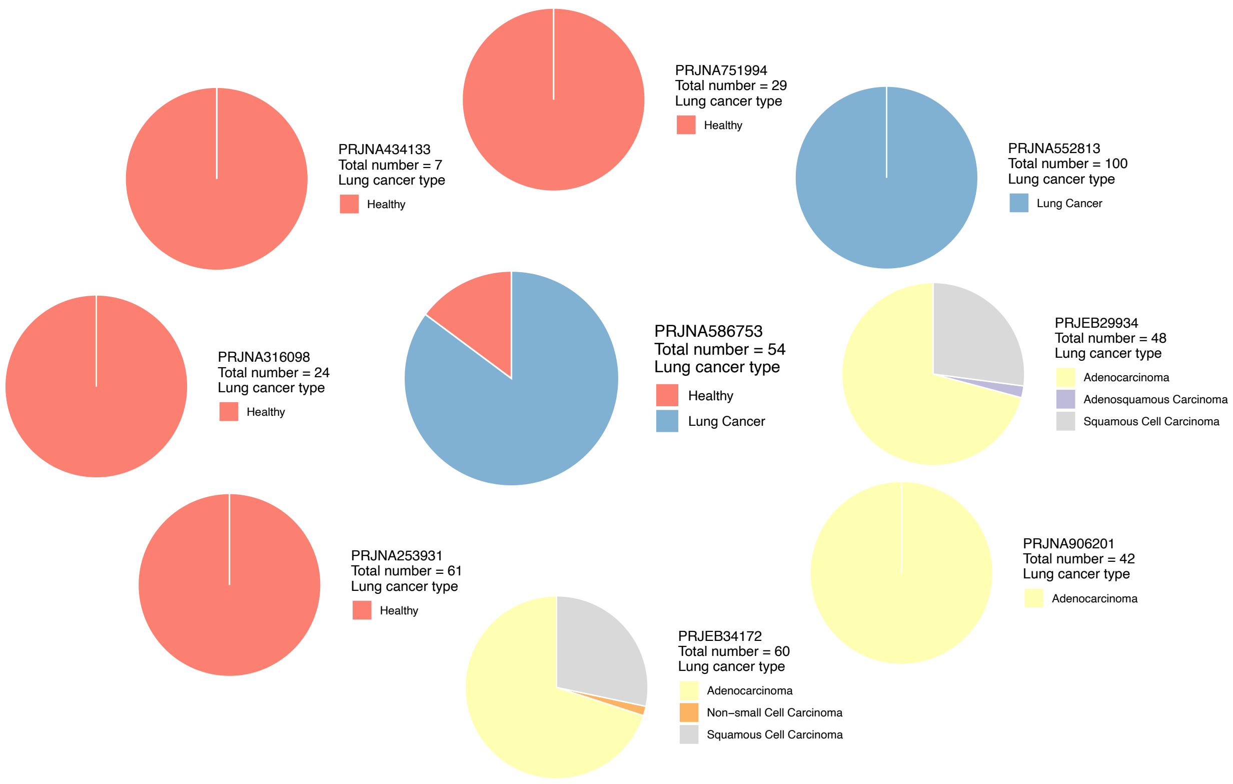

Fig. S3 Lung cancer subtype of participants with BAL samples in each cohort.

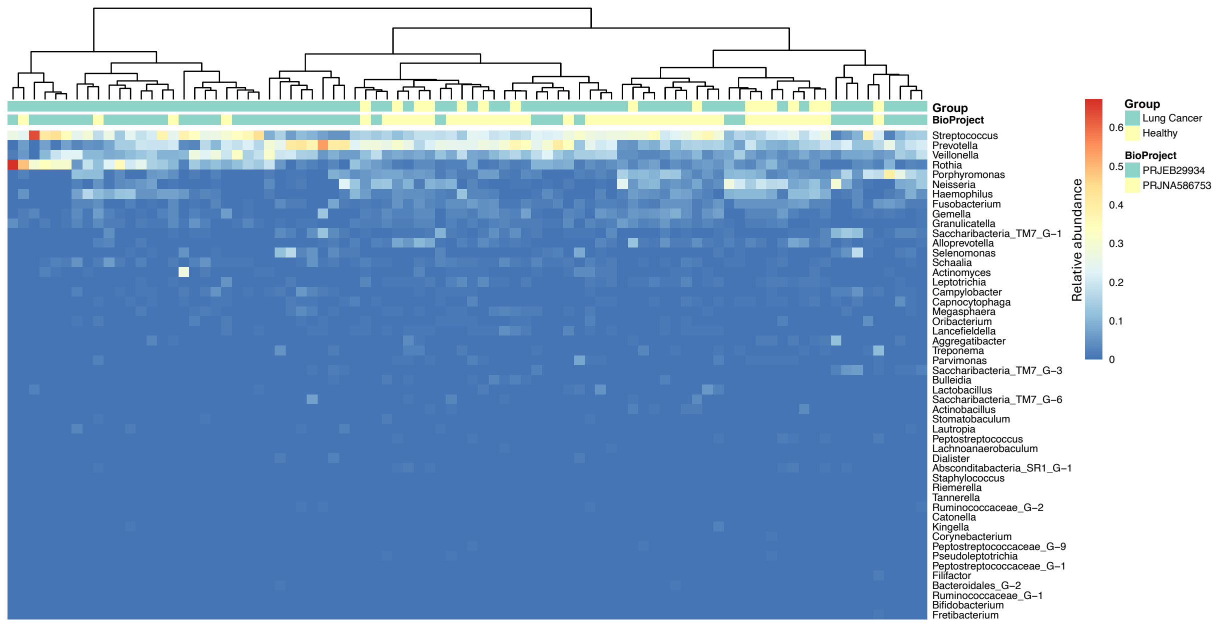

Fig. S4 The composition of the oral microbiota in healthy and lung cancer participants shown by the heatmap. Samples were clustered using the “ward.D2” method and Manhattan distance.

Case-matched lung tumor and saliva samples, n = 33 pairs

Taxa in tumor	Correlation coefficient	Taxa in saliva
Bacteriovorax	1	Bacteriovorax
Leucobacter	1	Leucobacter

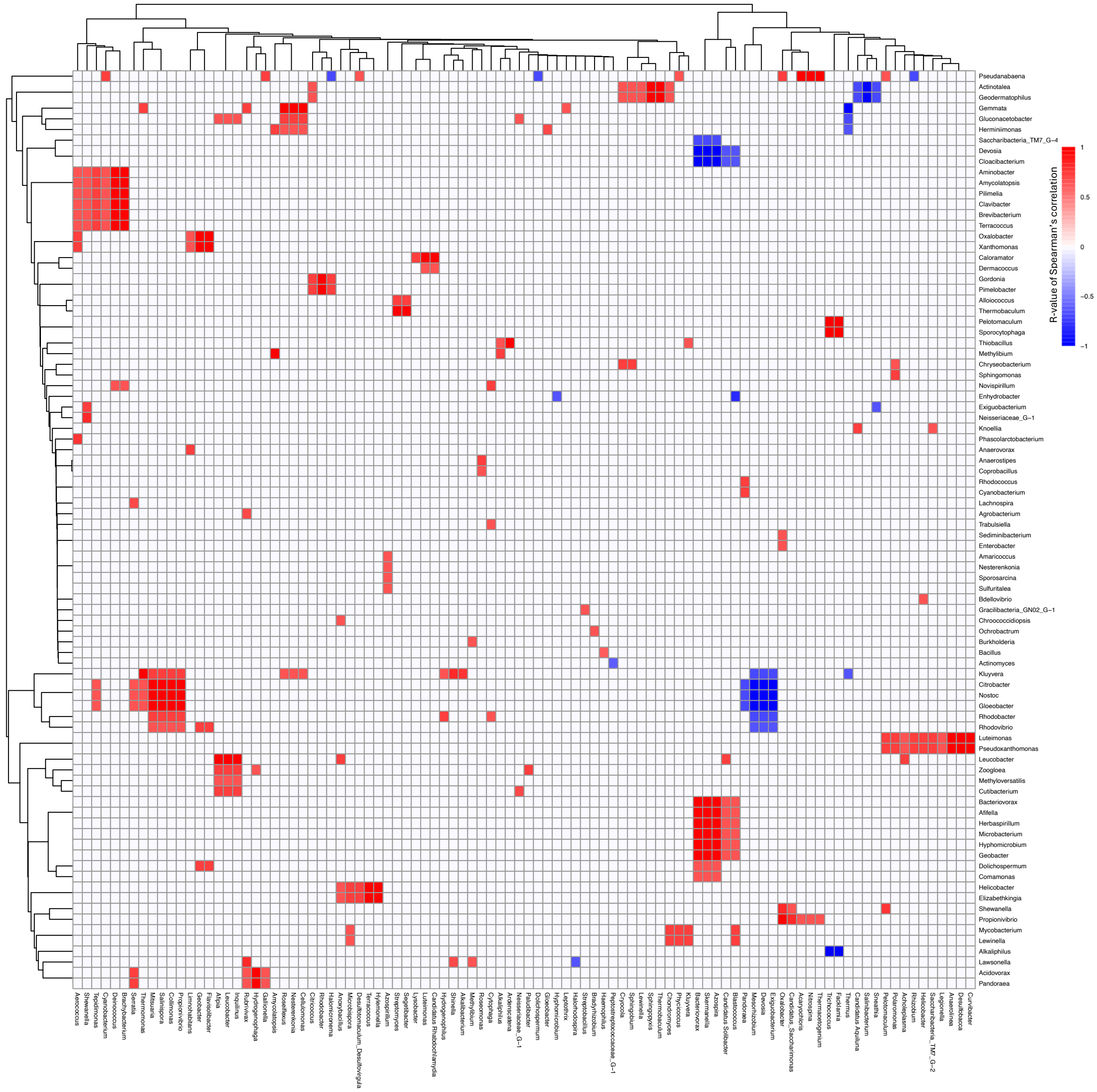

Fig. S5 Spearman's correlation between taxa in case-matched oral and BAL microbiota in the PRJNA586753 cohort. The correlation coefficient (R-value) and its significance (P-value) were calculated, with P-values adjusted using the Benjamini-Hochberg procedure via the 'adjust.p' function from the 'cp4p' package in R. In heatmap visualizations, R-values associated with adjusted P-values greater than 0.05 were replaced with zeros to exclude insignificant correlations. Taxa were clustered in the heatmaps based on the R-values of the Spearman's correlation using the 'pheatmap' function with default settings in R. As shown on the top panel, two genera in the oral microbiota were significantly associated with themselves in the BAL microbiota.

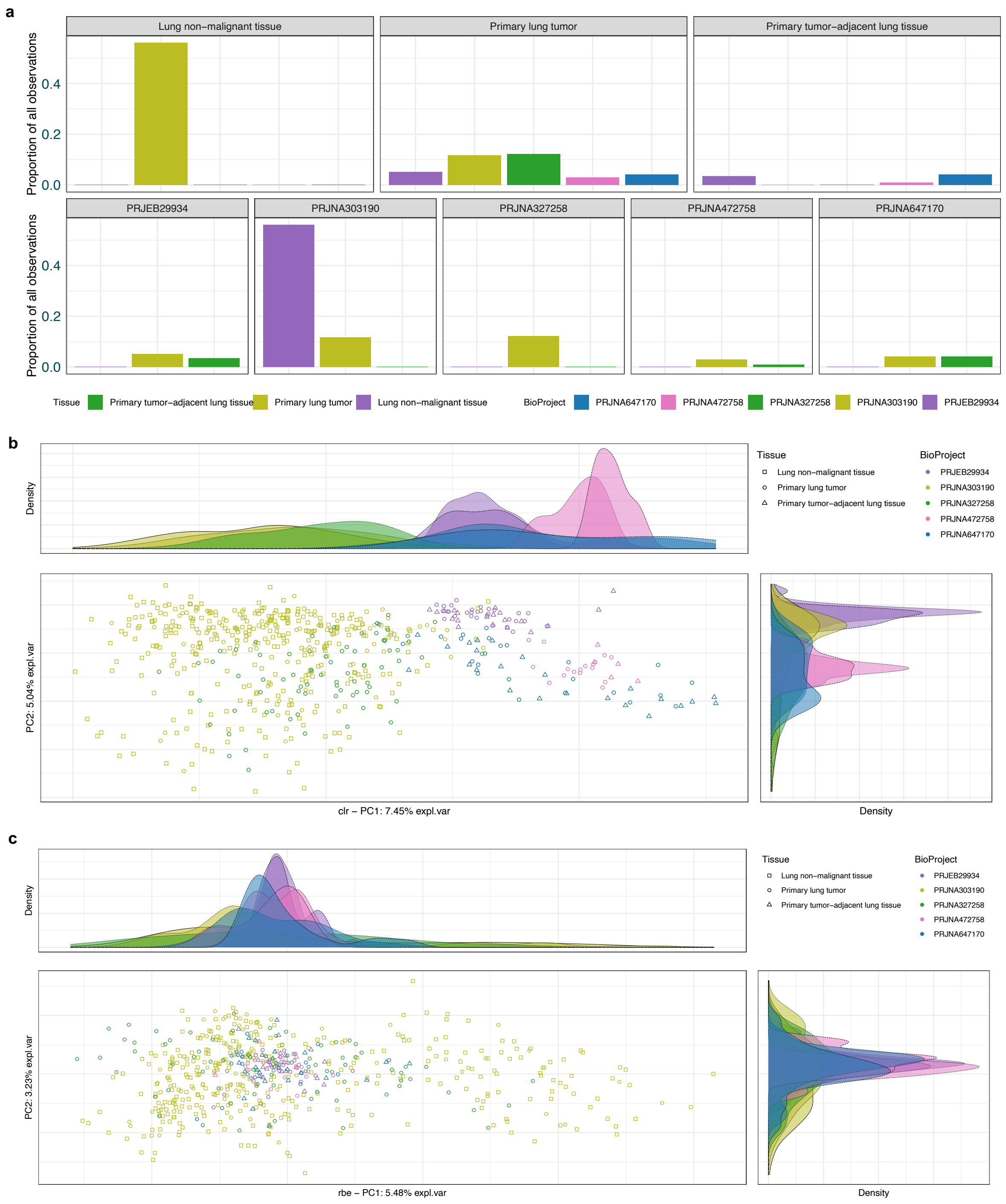

Fig. S6 Batch effect removal across lung tissue microbiota in different cohorts. (a) Number of tissue types in each cohort. The PCA plot shows the composition differences of the lung tissue microbiota among these cohorts before **(b)** and after **(c)** batch effect removal. Batch effects across cohorts were corrected using the 'MBECS' package in R, with the 'mbecCorrection' function set to method = "rbe" and type = "clr".

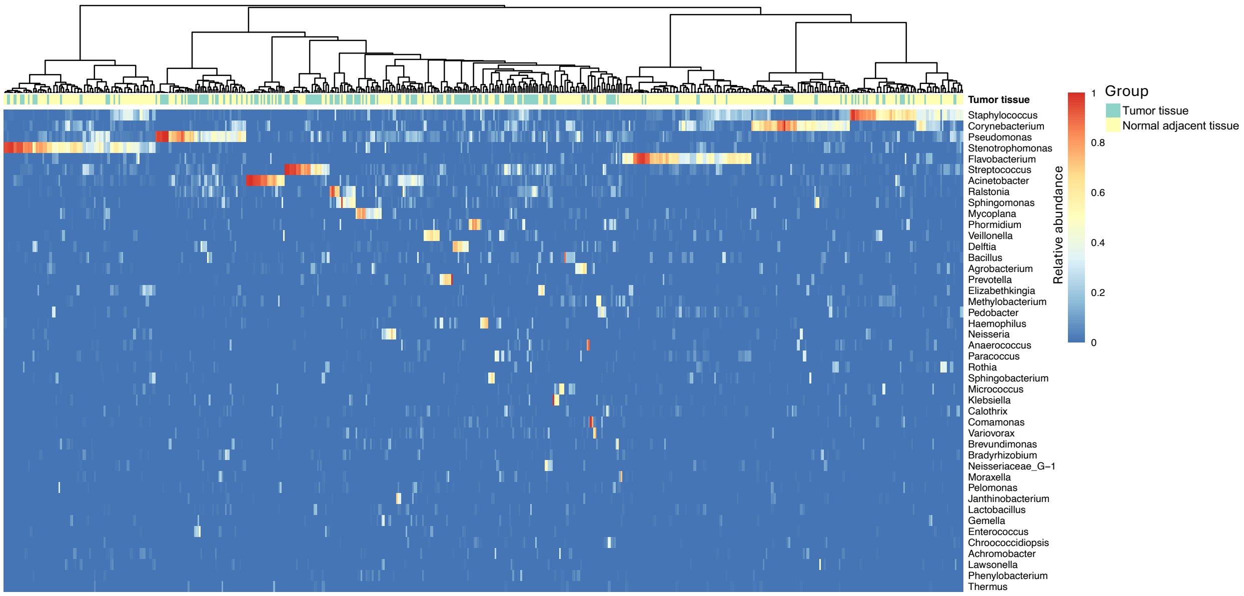

Fig. S7 The composition of the lung tissue microbiota in lung cancer participants shown by the heatmap. Samples were clustered using the “ward.D2” method and Manhattan distance.

a

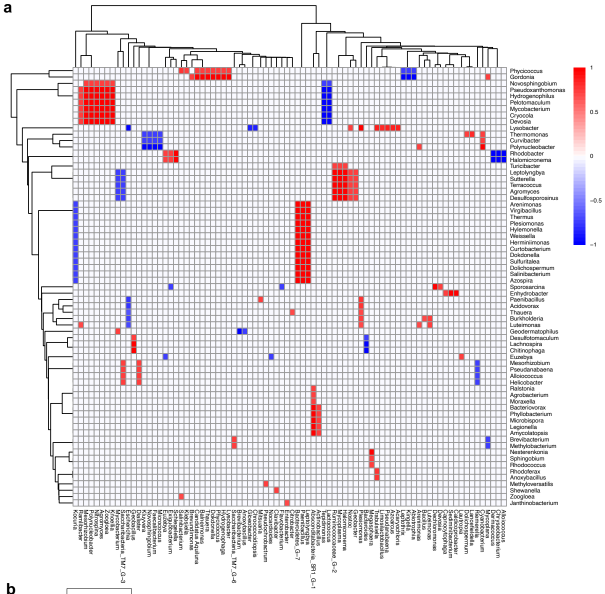

Case-matched BAL and oral samples, n = 45 pairs

4 out of 416 taxa in BAL correlates with itself in oral samples.

Taxa in Sailva	Correlation coefficient	Taxa in BAL
Streptobacillus	0.8257907	Streptobacillus
Deinococcus	0.5905204	Deinococcus
Oscillospira	0.7150969	Oscillospira
Roseburia	0.7865486	Roseburia

b

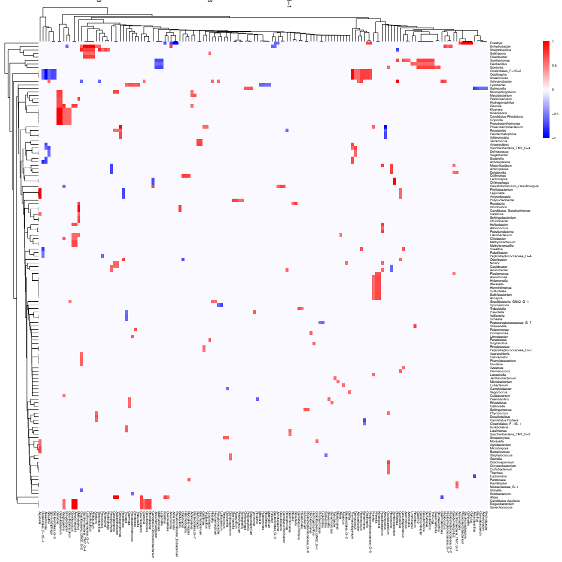

Case-matched lung tissue and oral samples, n = 27 pairs

2 out of 416 taxa in BAL correlates with itself in lung tissue samples.

Taxa in lung tissue	Correlation coefficient	Taxa in saliva
Luteimonas	0.7407056	Luteimonas
Phycicoccus	0.7205767	Phycicoccus

c

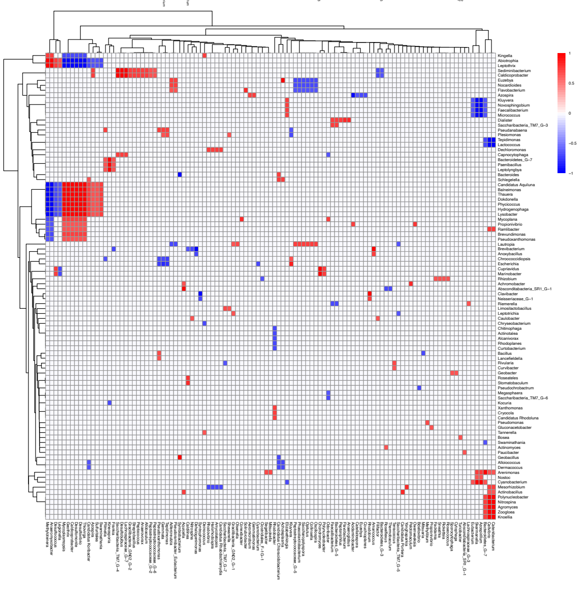

Case-matched lung tissue and BAL samples, n = 30 pairs

0 out of 416 taxa in BAL correlates with itself in lung tumors.

Fig. S8 Spearman's correlation between taxa in case-matched oral and BAL microbiota (a), oral and lung tissue microbiota (b), and BAL and lung tissue microbiota (c) in the PRJEB29934 **cohort.** The correlation coefficient (R-value) and its significance (P-value) were calculated, with P-values adjusted using the Benjamini-Hochberg procedure via the 'adjust.p' function from the 'cp4p' package in R. In heatmap visualizations, R-values associated with adjusted P-values greater than 0.05 were replaced with zeros to exclude insignificant correlations. Taxa were clustered in the heatmaps based on the R-values of the Spearman's correlation using the 'pheatmap' function with default settings in R. As shown on the right panel, genera in one microbiota significantly associated with themselves in the other microbiota are listed.
